# Supplementary material for: Splice-Junction-Based Mapping of Alternative Isoforms in the Human Proteome
Source: Cell Rep. Author manuscript; Available in PMC 2020 Jan 15. (PMC6961840; doi:10.1016/j.celrep.2019.11.026)
Supplement: 2 [file NIHMS1546469-supplement-2.zip › DF1/PXD006675/InferiorVenaCava/InferiorVenaCava_9_TRIM28_RSGAAHSAMCSLTAAAASAASGSPGPGEGSAGGEK.pdf]

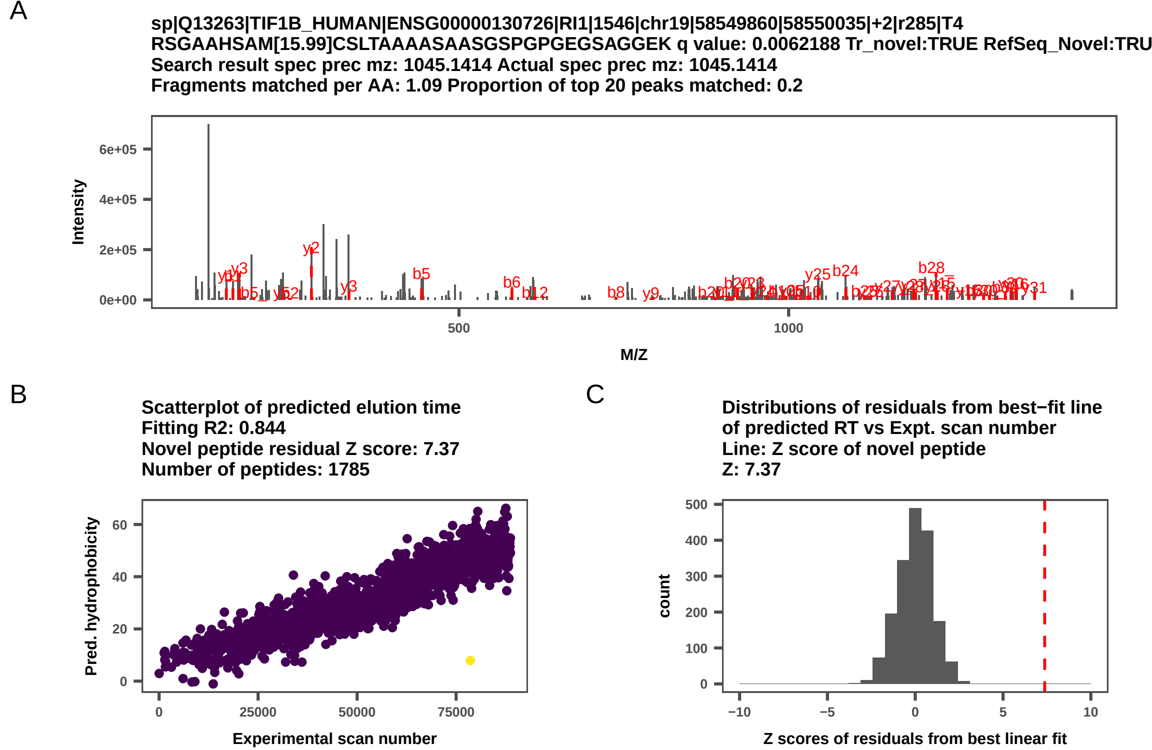
A
sp|Q13263|TIF1B_HUMAN|ENSG00000130726|RI1|1546|chr19|58549860|58550035|+2|r285|T4
RSGAAHSAM[15.99]CSLTAAAASAASGSPGPGEGSAGGEK q value: 0.0062188 Tr_novel:TRUE RefSeq_Novel:TRU
Search result spec prec mz: 1045.1414 Actual spec prec mz: 1045.1414
Fragments matched per AA: 1.09 Proportion of top 20 peaks matched: 0.2
Intensity
6e+05
4e+05
2e+05
0e+00
y2
y3
b5
b6
b12
b8
y9
y25
b24
b28
y31
500
1000
M/Z
B
Scatterplot of predicted elution time
Fitting R2: 0.844
Novel peptide residual Z score: 7.37
Number of peptides: 1785
Pred. hydrophobicity
60
40
20
0
0
25000
50000
75000
Experimental scan number
C
Distributions of residuals from best−fit line
of predicted RT vs Expt. scan number
Line: Z score of novel peptide
Z: 7.37
count
500
400
300
200
100
0
−10
−5
0
5
10
Z scores of residuals from best linear fit
